# Supplementary material for: Content-rich biological network constructed by mining PubMed abstracts
Source: BMC Bioinformatics. 2004 Oct 8;5:147. doi: 10.1186/1471-2105-5-147 (PMC528731; doi:10.1186/1471-2105-5-147)
Supplement: Additional File 2 — The original results of the above study (non-essential files are deleted to keep the file size under the limit set by BMC bioinformatics). [file 1471-2105-5-147-S2.bz2 › chilibotAdditionalFile2/dip05/243ID8921390E799/html/RAD51_UBE2I.html]

 


 **RAD51** and **UBE2I** 
  
Found 7 abstracts in PubMed, retrieved 05.  
 

 What does Google say? 
 PDF only 
| .edu only 

---

**Interactive relationship** (e.g. stimulation, inhibition, etc)

**Neutral relationship**- PCNA is mono ubiquitinated through RAD6 and RAD18, modified by lysine 63 linked multi ubiquitination which additionally requires MMS2, UBC13 and RAD5  [ **RAD51** ]  and is conjugated to SUMO by UBC9  [ **UBE2I** ] .  Ref: 12226657 Nature, 2002
- These findings emphasize that the IR1 2 domain is a useful tool for manipulating the nuclear localization of Ubc9  [ **UBE2I** ]  and perturbing the subcellular localization of SUMOs and or SUMOlated proteins, and they emphasize the important role of nuclear Ubc9  [ **UBE2I** ]  in the  **Rad51**  mediated homologous recombination pathway, possibly by modulating intracellular trafficking of  **Rad51** .  Ref: 11709548 J Biol Chem, 2002

**Non-interactive relationship** (e.g. studied together, co-existance, homology, etc.)

- Four major functional domains have been identified a DNA binding domain amino acids 1 85, a self association and UBC9  [ **UBE2I** ]  interacting domain amino acids 85 159, an RPA interacting domain amino acids 221 280, and a  **RAD51**  interacting domain amino acids 287 330.  Ref: 12372413 Biochem Biophys Res Commun, 2002
